# Supplementary material for: Content-rich biological network constructed by mining PubMed abstracts
Source: BMC Bioinformatics. 2004 Oct 8;5:147. doi: 10.1186/1471-2105-5-147 (PMC528731; doi:10.1186/1471-2105-5-147)
Supplement: Additional File 2 — The original results of the above study (non-essential files are deleted to keep the file size under the limit set by BMC bioinformatics). [file 1471-2105-5-147-S2.bz2 › chilibotAdditionalFile2/dip20/152ID8479754E547/html/CCND1_CDK2.html]

 


 **CCND1** and **CDK2** 
  
Found 643 abstracts in PubMed, retrieved 20.  
 

 What does Google say? 
 PDF only 
| .edu only 

---

**Interactive relationship** (e.g. stimulation, inhibition, etc)

**Inhibitory relationship**- However, the activity of cyclin E  **CDK2**  was decreased in a concentration and time dependent manner and the activity of cyclin D  [ **CCND1** ]  CDK4 was inhibited when serum starved synchronized cells were released from synchronization.  Ref: 12781329 Biochem Pharmacol, 2003
**Stimulatory relationship**- The bypass of TNF induced G1 arrest seems to be related to the increase in the stability of cyclin D  [ **CCND1** ]  bound CDK complexes, increasing the total amount of  **CDK2**  4 and 6 complexes and leading to a functional down titration of the p21 waf1 molecules.  Ref: 12761882 J Cell Biochem, 2003
**Neutral relationship**- We propose a mathematical model for the regulation of the G1 phase of the mammalian cell cycle taking into account interactions of cyclin D  [ **CCND1** ]  cdk4, cyclin E  **cdk2** , Rb and E2F.  Ref: 12695688 Cell Cycle< MedlineTA>Cell Cycle, 2003

**Non-interactive relationship** (e.g. studied together, co-existance, homology, etc.)

- Levels of cyclin D1  [ **CCND1** ] , cyclin E, CDK 2  [ **CDK2** ]  and CDK 4 were reduced in carcinomas from corticosterone treated rats.  Ref: 12807724 Carcinogenesis< MedlineTA>Carcinogenesis, 2003
- Positive staining for cyclin D1  [ **CCND1** ] , cyclin E, cyclin A, cyclin B1,  **cdk2** , cdk4, cdc2, p53, p21, and p27 was observed in 63%, 66%, 31%, 32%, 51%, 77%, 71%, 43%, 35%, and 60% of the 82 carcinomas, respectively.  Ref: 12792921 Hum Pathol, 2003
- In the lactacystin treated OLGcs, there was a dose dependent decrease in the number of cells incorporating bromodeoxyuridine and in the activity of the complexes cyclin D  [ **CCND1** ]  cdk4 and cyclin E  **cdk2** .  Ref: 12805303 J Neurosci, 2003
- Bcl  [ **CCND1** ]  2 antiproliferative effect in E1A c Ha ras transformants was not associated with alterations in  **Cdk2** , cyclin E and A contents.  Ref: 12722479 Tsitologiia, 2003
- In contrast, degradation of the AhR in HepG2 liver cancer cells resulted in decreased G0 G1 > S phase progression, and this was accompanied by decreased expression of cyclin D1  [ **CCND1** ] , cyclin E, cyclin dependent kinase 2  [ **CDK2** ]   **cdk2** , and cdk4.  Ref: 12761348 Mol Pharmacol, 2003
- Quantitative immunochemical evaluation of the expression of certain proteins controlling the cell cycle showed an increase in p27kip1, cyclin D  [ **CCND1** ] , and cdk4 positive cells, with a decrease in cyclin E and  **cdk2**  positive cells.  Ref: 12805303 J Neurosci, 2003
- EGF administration after PH also significantly affected the activity of the cyclin D1  [ **CCND1** ]  cdk4 and cyclin E  **cdk2**  complexes, mainly by changing their time progression it accelerated the increase in activity to 18h and caused a subsequent drop in activity after 34h.  Ref: 12876382 Cell Physiol Biochem, 2003
- Western blot analysis of G 1 phase regulatory proteins demonstrated that the protein levels of cyclin dependent kinase 2  [ **CDK2** ]   **Cdk2** , Cdk4, Cyclin E and Cyclin D3 were decreased after treatment with SCK6 but not those of Cdk6, Cyclin D1  [ **CCND1** ]  and D2.  Ref: 12706452 Eur J Pharmacol, 2003
- In a series of 23 surgical biopsies of grade II and III oligodendroglioma, cyclin D1  [ **CCND1** ] , E, A, B1, CDK4 6,  **CDK2** , Cdc2 and p27 Kip.1 have been studied by immunohistochemistry and Western blot.  Ref: 12873740 Neurosci Lett, 2003
- Overall, our results indicate that Rho kinase dependent stress fiber formation is required for sustained activation of the MEK ERK pathway and the mid G 1 phase induction of cyclin D1  [ **CCND1** ] , but not for other aspects of cdk4 or  **cdk2**  activation.  Ref: 12773570 Mol Cell Biol, 2003
- Western blotting analysis revealed that the protein levels of cyclin A, cyclin E, and cyclin dependent kinase CDK 2  [ **CDK2** ]  but not cyclin D1  [ **CCND1** ]  and CDK4 decreased after progesterone treatment, but those of CDK inhibitory proteins, p21 and p27, increased.  Ref: 12810531 Endocrinology< MedlineTA>Endocrinology, 2003
- These data identify c Myc, cyclin D1  [ **CCND1** ] , p21 WAF1 CIP1 and cyclin E  **Cdk2**  as central components of estrogen regulation of cell cycle progression and hence as potential downstream targets that contribute to the role of estrogen in oncogenesis.  Ref: 12790780 Endocr Relat Cancer< MedlineTA>Endocr Relat Cancer, 2003
- Arsenic increased expression of the P21 protein and decreased levels of cyclin A, cyclin B1 and cyclin D1  [ **CCND1** ] , but expression of  **CDK2** , CDK4, CDK6, and cyclin E were not affected.  Ref: 12883267 Pancreas, 2003
- Binding of these CKI s to both the cyclin D1  [ **CCND1** ]  CDK 4 complex and the cyclin E CDK 2  [ **CDK2** ]  complex were increased and the kinase activities of these complexes were reduced with increasing dietary corticosterone.  Ref: 12807724 Carcinogenesis< MedlineTA>Carcinogenesis, 2003
- LY CDK inhibitor treated cells also displayed diminished pRb dephosphorylation on  **CDK2**  and CDK4 specific sites, retinoblastoma protein cleavage, and down regulation of cyclin D  [ **CCND1** ] .  Ref: 12702569 Cancer Res, 2003
- These results suggest that the suppression of cell cycle progression in the G1 phase by 5GG was mediated in MCF 7 cells, at least in part, by either the inhibition of cyclin D  [ **CCND1** ]  CDK4 and cyclin E  **CDK2**  activity or the induction of the CDK inhibitors p27 Kip and p21 Cip.  Ref: 12781329 Biochem Pharmacol, 2003
- Monensin decreased the levels of  **CDK2** , CDK6, cdc2, cyclin A, cyclin B1, cyclin D1  [ **CCND1** ]  and cyclin E proteins but did not alter CDK4 protein.  Ref: 12792794 Int J Oncol, 2003
